# Edaravone: A Novel Possible Drug for Cancer Treatment?

**DOI:** 10.3390/ijms25031633

**Published:** 2024-01-29

**Authors:** Elisa Duranti, Nicoletta Cordani, Chiara Villa

**Affiliations:** School of Medicine and Surgery, University of Milano-Bicocca, 20900 Monza, Italy; e.duranti@campus.unimib.it (E.D.); nicoletta.cordani@unimib.it (N.C.)

**Keywords:** edaravone, oxidative stress, cancer

## Abstract

Despite significant advancements in understanding the causes and progression of tumors, cancer remains one of the leading causes of death worldwide. In light of advances in cancer therapy, there has been a growing interest in drug repurposing, which involves exploring new uses for medications that are already approved for clinical use. One such medication is edaravone, which is currently used to manage patients with cerebral infarction and amyotrophic lateral sclerosis. Due to its antioxidant and anti-inflammatory properties, edaravone has also been investigated for its potential activities in treating cancer, notably as an anti-proliferative and cytoprotective drug against side effects induced by traditional cancer therapies. This comprehensive review aims to provide updates on the various applications of edaravone in cancer therapy. It explores its potential as a standalone antitumor drug, either used alone or in combination with other medications, as well as its role as an adjuvant to mitigate the side effects of conventional anticancer treatments.

## 1. Introduction

Despite extensive efforts to find new treatment approaches, cancer remains a dominant cause of death and a significant burden on healthcare systems around the world [1]. Cancer refers to a group of disorders characterized by uncontrolled cell growth. This growth is caused by the activation of proto-oncogenes and/or the inactivation of tumor-suppressor genes [2,3]. The process of transforming a normal cell into a cancerous one involves several steps. This transformation leads to an increased rate of cell division, the ability to escape signals that inhibit cell growth and death, the initiation of angiogenesis (formation of new blood vessels), and ultimately, the activation of programs that enable the invasion of surrounding tissues and metastasis [4,5,6]. Throughout cancer progression, tumors tend to exhibit notable heterogeneity, resulting in diverse cell populations with distinct molecular characteristics and varied responses to therapies [7]. This heterogeneity can manifest spatially and temporally and plays a crucial role in the development of resistance phenotypes, which are influenced by selective pressures exerted during treatment administration. Therefore, a comprehensive understanding of these intricate processes is essential for the development of targeted and efficacious treatments [7].

The increasing global burden of cancer highlights the pressing need to develop new strategies that can prevent these diseases from occurring and explore new therapeutic targets while identifying innovative treatments. The main focus for achieving complete cancer remission is typically centered around surgically removing the tumor and any associated metastases [8,9,10,11]. Despite various screening programs aimed at reducing tumor incidence, numerous cancer cases are diagnosed at advanced stages, making surgical removal impossible. Current cancer therapies face several challenges related to their selectivity, specificity, complexity, side effects, and most importantly, drug resistance [12,13]. Considering these factors, it is crucial to continue making ongoing efforts to reassess therapeutic approaches and develop more efficient cancer medications that can overcome these existing obstacles [14]. In such scenarios, drug repurposing, sometimes referred to as drug repositioning, offers an alternative to de novo drug development since it involves the identification of a new therapeutic use for an existing drug approved for a different indication [15,16]. Drug repurposing offers advantages over traditional drug discovery, including reduced costs and time, increased efficiency, and lower risk of failure [17]. Drug repurposing offers two major benefits: availability of preclinical data on the existing drug and further knowledge about its clinical features, including pharmacokinetics, pharmacodynamics, and toxicity profiles [18]. In this context, edaravone, a free radical scavenger prescribed for the management of acute cerebral infarction and amyotrophic lateral sclerosis (ALS), has been recently investigated also for the treatment of cancer, given its antioxidative and anti-inflammatory properties [19]. Herein, we will update the latest developments in the use of edaravone as a potential treatment for cancer. Specifically, we will examine its potential as a standalone direct antitumor medication and its capacity to synergize with other therapies. Furthermore, we will explore its potential as an adjuvant support to mitigate the adverse side effects associated with existing anticancer treatments.

## 2. Conventional Therapies for Cancer

**Figure 1:**
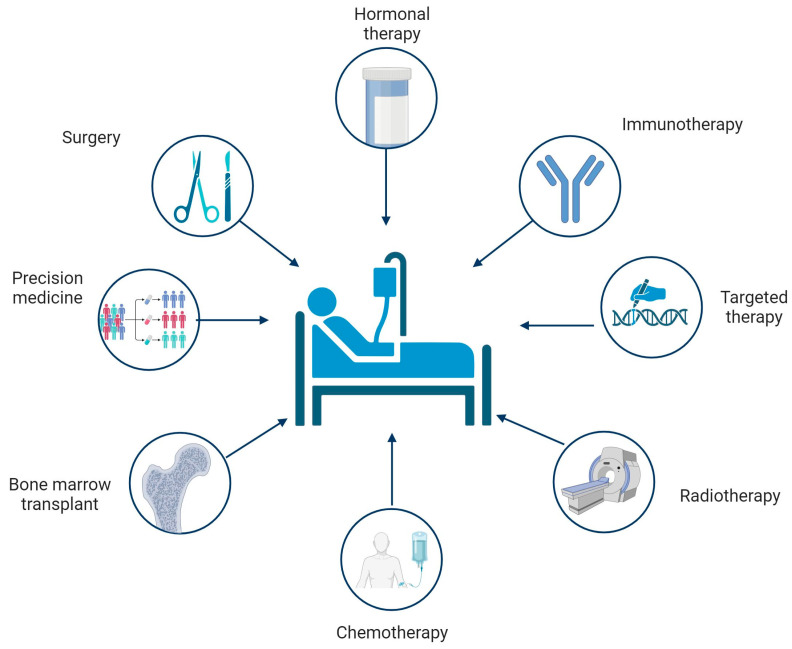
Schematic figure of current cancer therapies. Created with BioRender.com (accessed on 17 January 2024).

There are various treatment options for cancer, and the choice of a therapeutic plan is determined by oncologists based on the patient’s oncological condition (Figure 1) [20,21]. Cancer treatment depends on two critical factors: the stage and type of cancer. When the disease is detected at an early stage, the treatment may strive to achieve a complete cure. In other cases, the goal is to halt cancer progression, preventing further growth and spread. It is important to highlight that the chosen treatment approach may change during therapy [21]. Oncologists regularly monitor the patient’s response to treatments, modifying therapy as needed to maximize success [22]. In recent years, there have been significant advances in oncology research resulting in novel therapeutic options, such as targeted therapies and immunotherapies. These therapeutic strategies focus on specific targets in the cancer growth process and its interaction with the patient’s immune system, leading to more personalized and effective treatments. With such a wide range of therapeutic options available, oncologists must carefully evaluate each alternative to maximize benefits while minimizing side effects. The current range of cancer treatments in clinical practice encompasses several strategies that are briefly outlined in Table 1. Ongoing scientific research continues to broaden the therapeutic horizon, offering hope for more effective and personalized cancer treatments.

### 2.1. Surgical Removal

Surgery represents one of the pillars of clinical cancer management and it is frequently the first line of treatment for localized solid tumors by removing cancer tissue and lymph nodes. If the cancer is in its early stages and has not spread to other parts of the body, surgery can be curative. However, curative surgery is not always possible, as most cancer cases are diagnosed at locally advanced or even metastatic stages. Furthermore, recurrence rates after resection remain high due to incomplete removal of cancer cells or systemic inflammation caused by surgical trauma and post-operative complications, resulting in the release of pro-inflammatory mediators [23]. Amputation is often the primary treatment for many limb tumors, as it can be challenging to perform a complete resection while preserving vital nerves and arteries. This can lower patients’ quality of life [24].

The main objective of advancing oncological surgery is to minimize invasiveness, thereby decreasing perioperative complications and post-operative side effects, while simultaneously enhancing patients’ quality of life without compromising the effectiveness of anticancer treatment [25]. In cases of gastric and colorectal cancer, laparoscopic surgery has demonstrated comparable anticancer efficacy to open surgery, with a decreased incidence or severity of complications [26,27]. However, further evidence is required to justify the routine use of laparoscopic gastrectomy [28]. Endoscopic surgery has been used in specialist centers to treat selected patients with early oesophageal, gastric, and colorectal cancer, including T1a stomach cancer without lymph node metastases [29,30]. A number of minimally invasive tumor ablation techniques have been developed as alternatives to open surgery, which include physically excising the entire tumor from the patient along with a layer of surrounding tissue as a safety precaution. These virtual surgical techniques, which deliver lethal temperatures, such as hyperthermal radiofrequency ablation and hypothermal cryotherapy or caustic chemicals, like absolute ethanol and acetic acid with resulting tissue necrosis, kill the tumor in situ instantaneously without actually removing the tumor [31].

### 2.2. Chemotherapy

Chemotherapy is a therapeutic approach that aims to eradicate tumor cells or reduce the growth of tumor cells while preserving normal cells. This treatment is typically administered in advanced stages when surgical resection is not feasible to reduce tumor mass and impede its progression. Moreover, it could be applied post-operatively to eliminate residual malignant cells or prevent the risk of recurrence. Chemotherapeutic agents kill tumor cells through a variety of mechanisms, such as activating intrinsic and extrinsic pathways of apoptosis, inducing cell cycle arrest by activating the p53 gene, stimulating kinase inhibition, activating the autophagy pathway, alkylating DNA, inhibiting DNA or RNA synthesis, disrupting microtubules, generating reactive oxygen species (ROS), and inhibiting topoisomerases. These mechanisms are intended to modify aberrant cellular proliferation and metabolism, which are cardinal features of malignancies [32].

Chemotherapy can be administered through various methods, including oral, intravenous, injection, intrathecal, intra-arterial, and intraperitoneal, targeting rapidly dividing cells [32,33]. Chemotherapy patients frequently experience multidrug resistance due to a variety of mechanisms activated by cancer cells, such as augmented xenobiotic metabolism, improved drug efflux, growth factors, enhanced DNA repair capacity, and genetic factors (gene mutations, epigenetic modification, and amplifications) [34]. These complex processes are often interrelated and can present significant challenges in the treatment of cancer patients. As such, it is crucial to develop new, effective strategies to combat multidrug resistance to improve patient outcomes and overall treatment success rates [35]. Chemotherapy is a fundamental component of oncological treatment; however, it is accompanied by a range of side effects that can be distressing for patients. These include alterations in taste perception, decreased appetite, nausea and vomiting, fatigue, alopecia, xerostomia, and constipation. Despite the benefits of chemotherapy, the deleterious effect of these side effects on patients’ quality of life necessitates the use of supportive care measures. Chemotherapy is a cancer treatment that is associated with a variety of side effects, including cognitive impairment and peripheral neuropathy [36]. Chemotherapy-induced cognitive impairment is a prevalent and debilitating side effect that affects more than 20% of breast cancer patients [36,37,38,39]. Taxane-induced peripheral neuropathy (TIPN) is a significant and common side effect that arises from the use of neurotoxic taxane agents in cancer treatment [40]. Patients with TIPN experience pain, and the treatment dosage may be limited as a result. As survival rates for cancer patients continue to improve, the incidence of TIPN is expected to rise [41,42]. Improved delivery could make these and other therapies more effective. For example, mesenchymal stromal cells (MSCs) have been demonstrated to be promising drug delivery vectors for treating cancer and other diseases. The use of MSCs for transporting and delivering anticancer drugs has potential advantages over free-form drugs. This is because MSCs can protect the drug from degradation before reaching the target cancer cells. Additionally, MSCs can integrate into the tumor stroma, enhancing local drug concentrations while decreasing systemic toxicity. Improved delivery could make these and other therapies more effective [43]. This approach has been validated in vitro and in vivo in a mesothelioma model [44], demonstrating its effectiveness.

### 2.3. Radiotherapy

Radiotherapy treatment is a medical procedure that employs ionizing radiation to eradicate tumor cells. This is achieved either directly through inducing DNA damage or indirectly through the generation of ROS, which can lead to cancer cell apoptosis. The use of radiotherapy is a widely accepted and effective approach for the treatment of cancer and is often employed either as standalone therapy or in combination with other treatments, such as chemotherapy or surgery. The success of radiotherapy depends on various factors such as the type of cancer, its stage of development, and the overall health of the patient [45]. The optimal delivery of radiotherapy is based on a balance between maximizing the dosage to the tumor and minimizing exposure to normal tissue since the mechanism of inducing death is not specific to tumor cells [46]. External beam radiotherapy (EBRT), brachytherapy, and intravenous radioisotopes can be used to treat tumors based on their type and location [47]. Radiation therapy can be used for both curative and palliative purposes, depending on the therapeutic situation and the symptoms experienced by the patient. The effectiveness of treatment and the likelihood of developing resistance depend on factors such as the growth rate of the cells and the degree of differentiation. Additionally, the total amount of radiation exposure and the frequency of the treatments received can impact the outcome of the therapy [48]. Radiotherapy’s side effects on normal tissues might be categorized as acute (early), consequential, or late [49]. It is worth noting that radiotherapy also has consequences for the central nervous system (CNS) [50,51], the most significant are radionecrosis and cognitive dysfunction/leukoencephalopathy [51,52].

### 2.4. Hematopoietic Stem Cell Transplantation (HSTC)

This medical procedure, commonly known as bone marrow transplantation, includes the administration of healthy hematopoietic stem cells to patients with defective or depleted bone marrow [53]. HSTC is the most effective therapeutic option to treat bone marrow-derived diseases, hematological malignancies, metabolic disorders, and immunological deficiencies [54,55,56]. In severe hematological tumors, HSTC is used in combination with high-dose chemotherapy or radiotherapy to consolidate remission and offer a long-term treatment [57]. Transplants can be autologous when the stem cells come from the same patient, allogeneic when the cells come from a compatible donor, or syngeneic when the patient has an identical twin [54,55,56,57].

### 2.5. Hormone Therapy

Hormone therapy is a treatment that involves the use of synthetic hormones or drugs to prevent the growth of tumor mass by blocking the hormones required for cell survival. It is mainly used to treat certain types of breast and prostate cancers that depend on hormones to grow.

Hormone receptor-positive (HR+) human epidermal growth factor receptor type 2 negative (HER2−) breast cancer (BC) is the most common subtype, accounting for approximately 70% of all BC cases [58]. It has been estimated that around 20–30% of patients with this type of tumor will experience relapse and develop metastatic disease. The median overall survival for patients with HR+/HER2− metastatic BC is 46 months [59]. The use of CDK4/6 inhibitors (palbociclib, abemaciclib, or ribociclib) in combination with endocrine therapy has resulted in significant changes in clinical practice. These treatments are currently recommended as first-line therapies for all metastatic breast cancer patients without visceral crisis. Prostate cancer (PCa) is one of the most prevalent types of tumors and is the second leading cause of cancer-related death among men.

Following definitive treatments, such as radical prostatectomy (RP) and external beam radiation therapy (EBRT), patients may experience a biochemical recurrence (BCR) within 10 years, affecting 20–50% of patients [60]. Clinical trials have explored the use of systemic therapy for androgen axis suppression in BCR. However, it is important to consider the toxicity of ADT, particularly for asymptomatic patients who are unwilling to undergo therapies with significant toxicity. ADT has been reported to have modest survival benefits, especially for those at lower risk of clinical progression. However, long-term use of ADT is associated with several side effects, such as fatigue, impaired sexual and hormonal quality of life, anemia, osteoporosis, diabetes mellitus, and cardiovascular morbidity [61,62,63,64,65].

### 2.6. Immunotherapy

Immunotherapy is a treatment that aims to enhance the immune system’s ability to recognize and attack cancer cells [66,67]. It can also mitigate the side effects associated with other cancer treatments [68]. Several approaches fall under the umbrella of immunotherapy, including immune checkpoint inhibitors, which block specific checkpoints to boost the immune response against cancer cells. Chimeric antigen receptor (CAR) T-cell transfer therapy is a strategy that enhances the ability of T cells to fight cancer by selecting and modifying the most efficient immune cells against cancer. Monoclonal antibodies, designed to target specific antigens on cancer cells, help the immune system recognize and destroy them. Therapeutic vaccines are also being studied to improve the immune system’s response to cancer [69,70]. However further studies are needed to fully understand the potential and mechanisms of these strategies.

### 2.7. Targeted Therapy

Targeted therapy aims to deliver drugs to specific genes or proteins that are found only in cancerous cells or in the tissue microenvironment that is driving tumor growth. It is often used in combination with other types of cancer treatment, such as chemotherapy [13,21,71]. Targeted therapy involves developing drugs that inhibit cancer proliferation, promote cell cycle regulation, induce apoptosis or autophagy, or deliver harmful substances only to cancer cells to eliminate them [72]. Oral small-molecule drugs or monoclonal antibodies are used in targeted cancer therapy [73,74]. This treatment can improve overall survival and progression-free survival. However, patients can develop resistance and eventually succumb to tumor progression [75,76]. The successful blockade of cancer cell signaling pathways by targeted therapies exerts significant selective pressure on cells to find alternative mechanisms to evade the response to the drug. The loss of checkpoint control genes, which actively monitor and maintain genomic integrity, increases the likelihood of the global emergence of drug-resistant cells [77].

### 2.8. Precision Medicine

Precision medicine has transformed the field of oncologic biomarkers, medication discovery, and drug development. It has also significantly improved cancer patient outcomes by adopting a more personalized approach [78]. Precision medicine in oncology involves rapidly evolving methods for cancer treatment that utilize molecular profiling to identify specific mutations or alterations that can be targeted with precision medicine drugs. The goal is to maximize clinical efficacy and response, minimize side effects, and reduce economic burden. Significant progress has been made in melanoma, breast, and lung cancer [79].

Advanced technologies have recently emerged, revolutionizing the field of personalized medicine. These include liquid biopsy and companion diagnostics, accompanied by the development of new prognostic and predictive markers, such as minimal residual disease. Medical societies’ clinical practice guidelines play a key role in harmonizing cancer care. Predictive biomarkers are tested for in newly diagnosed advanced-stage non-small-cell lung cancer (NSCLC) specimens [80]. Predictive biomarker testing is essential in standardizing cancer care. For instance, when a patient is diagnosed with advanced-stage NSCLC, their samples are examined for these biomarkers [80]. The evaluation checks for various genetic alterations, include epidermal growth factor receptor (EGFR) mutations, ALK fusions, ROS1 fusions, BRAF V600E, NTRK fusions, RET fusions, and MET exon 14 skipping alterations, as well as PD-L1 immunohistochemistry (IHC). This is crucial because highly effective targeted therapies have been approved for these specific targets [81], while tumor mutational burden (TMB) is approved for first-line immune checkpoint inhibition in lung cancer patients [82].

Several mutations have been identified in breast tumors that can be targeted for therapy in breast cancer treatment. Advances in omics technologies have led to more precise strategies for precision therapy. The development of next-generation sequencing technologies has raised hopes for precision medicine treatments in breast cancer and triple-negative breast cancer. Targeted therapy approaches, such as immune checkpoint inhibitors (ICIs) [83], EGFRi [84], and PARPi [85], are potential treatment options for BC and TNBC.

Recently, advances in multi-omics analyses, such as genomics, transcriptomics, proteomics, metabolomics, radiomics, etc., together with advances in data interpretation technology, have enabled researchers to identify and understand the various biological processes responsible for causing cancer. This has greatly improved the clinical management of tumors, particularly melanoma, which is one of the most aggressive forms of human cancer [86].

Precision medicine is a medical approach that aims to identify the most appropriate treatment for individual patients with colorectal cancer. This approach considers the molecular characteristics and specific vulnerabilities of the patient’s disease. It also takes into account the patient’s overall condition and ability to tolerate a combination of chemotherapy, immunotherapy, and targeted therapies. In particular, druggable RAS (KRAS, NRAS), BRAF, and mismatch repair (MMR) status are assessed to determine the most effective treatment options [87].

There are currently two test tools available to assess homologous recombination repair defects (HRDs), Myriad MyChoice and FoundationOne CDX. Myriad MyChoice utilizes next-generation sequencing (NGS) to generate a genomic instability score (GIS). The score is calculated by evaluating the loss of heterozygosity (LOH), telomeric allelic imbalance (TAI), and large-scale transitions (LST). This test is considered a companion diagnostic test for HRDs [71].

Several clinical trials have demonstrated that patients with advanced ovarian cancer who were treated with a PARPi, with or without bevacizumab, as a maintenance therapy following platinum-based therapy showed improved progression-free survival (PFS). This benefit was more significant in patients with homologous recombination deficiency (HRD), as determined by the Myriad myChoice test. The European Network for Gynaecological Oncology Trials (ENGOT) has recently sponsored the European HRD ENGOT initiative to assess various academic HRD tests developed by seven academic research laboratories [71].

## 3. Oxidative Stress in Cancer

**Figure 2:**
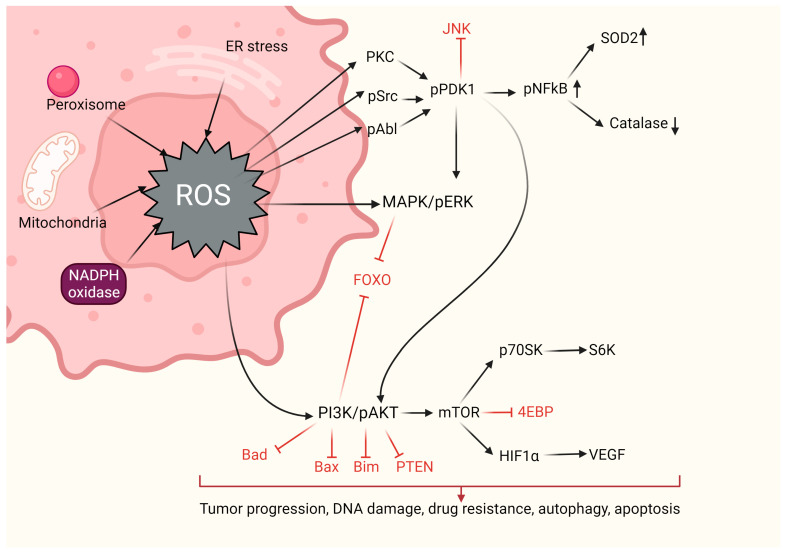
Main cellular signaling pathways involved in oxidative stress. Created with BioRender.com (accessed on 17 January 2024).

ROS are molecules containing oxygen that are continuously produced as byproducts during various biological processes, such as cellular respiration and energy generation. These ROS can be generated internally within the body or in response to external factors like ionizing radiation, air pollution, or harmful substances. The most notable ROS include the superoxide radical (O2·−), the hydroxyl radical (HO·), and hydrogen peroxide (H_2_O_2_) [88]. While ROS are necessary for the well-being and survival of organisms, an excessive amount of them can cause damage to cells, leading to a condition known as oxidative stress. Oxidative stress has been linked to several health issues, including neurodegenerative disorders, heart disease, diabetes, and various other ailments [89,90,91,92,93]. Hence, it is vital to regulate ROS levels to maintain cellular balance and prevent cellular damage and associated illnesses [94] (refer to Figure 2).

In the context of cancer, there is evidence that the production of ROS within tumor cells inactivates PTEN, leading to heightened PI3K/AKT signaling and cell proliferation. In addition, ROS can interfere with the function of the phosphatase Cdc14B, leading to the activation of cyclin-dependent kinase 1 (Cdk1), which facilitates the progression of the cancer cell cycle [79,88]. Hypoxia-inducible factor (HIF), a crucial gene regulated by ROS, triggers the activation of PDK1, subsequently initiating AKT and inhibiting the tuberous sclerosis complex (TSC). This, in turn, downregulates mTOR, a vital regulator of cell growth that governs processes such as mRNA translation, ribosome synthesis, autophagy, and cell metabolism [89,90]. The MAPK/ERK1/2 pathways, stimulated by growth factors and K-Ras signaling, drive increased cellular proliferation in cancer cells. Moreover, H_2_O_2_ has been identified as a factor that activates ERK1/2 and the pro-survival PI3K/AKT signaling pathway, thereby enhancing proliferation [54]. Extensive research in leukemia, melanoma, breast, and ovarian cancer has demonstrated various roles of ERK1/2, including cell survival, anchorage-independent growth, and cell motility [69,70,71,72,73]. The AKT pathway contributes to cell survival through the inactivation of pro-apoptotic factors and transcription factors via phosphorylation. AKT can be activated by H_2_O_2_ production derived from the epithelial growth factor (EGF), as observed in ovarian cancers [74]. Furthermore, the oxidation and inactivation of negative regulators of PI3K/AKT signaling, such as PTEN and PTP1B, promote cell survival. H_2_O_2_ has also been found to reversibly deactivate the tumor suppressor PTEN in various types of cancer [75,76]. Additionally, tumor suppressor genes encode proteins that function as antioxidants. For instance, p53 can regulate the expression of several antioxidant enzymes, including catalase, SOD2, and GPX1, thereby reducing ROS accumulation. However, frequent loss or mutation of p53 in most cancers leads to ROS accumulation and the promotion of pro-tumorigenic signaling [77]. As we mentioned in the previous paragraphs, tumors are progressive diseases, and in their more advanced stages in malignant cancers, they exhibit a distinguishing feature known as tumor metastasis. This process involves the spread of cancer cells from the primary tumor to neighboring tissues and distant organs and is, unfortunately, the leading cause of both morbidity and mortality among cancer patients [95,96]. Metastatization is a complex and multifaceted event, driven by a combination of factors. It occurs as a result of the cumulative genetic mutations within cancer cells and the intricate interplay between non-malignant and malignant cells. This complex interaction is often characterized by a dynamic communication between various transcription factors, including NF-ĸB, ETS-1 (ETS proto-oncogene 1, transcription factor), Twist, Snail, AP-1, and Zeb (zinc finger E-box binding homeobox). Additionally, metalloproteases, such as MMP-2 and MMP-9, as well as chemokines and cytokines, like transforming growth factor beta (TGF-β), further contribute to the metastatic process. During carcinogenesis, high levels of ROS, increased by both tumor-associated and environmental factors, enhance metastasis by the activation of the PI3K/Akt/mTOR and MAPK signaling pathways which, in turn, lead to the downstream activation of SNAIL, MMP-2, and MMP-9 enzymes, promoting the transition from an epithelial to a mesenchymal state, known as epithelial-to-mesenchymal transition (EMT). EMT is the primary driver of tumor metastasis, where epithelial cells lose their natural polarity and their ability to adhere to neighboring cells and gain enhanced mobility [97]. ROS have been shown in numerous studies to be a primary cause of EMT. By regulating the expression of MMP-9 and uPA (urokinase-type plasminogen activator), TGF-β1 promotes cell invasion and migration via ROS-based pathways [98,99,100,101,102]. ROS can also enhance tumor migration by upregulating cathepsin activity and hypoxia-mediated MMP production [101,102]. Pro-inflammatory factors, like tumor necrosis factor-alpha (TNF-α), TGF-β, interleukin-6 (IL-6), and interleukin-1 (IL-1) are produced by both cancer and non-cancer cells, and they activate NFκB and signal transducers and activators of transcription-3 (STAT3), triggering the EMT trans-differentiation program and the activity of matrix modifiers [103]. Another study emphasized the significance of ROS generation in TGF-β1-induced EMT in MDAMB-231C and MCG-10A cell lines, which is dependent on NADPH oxidase 4 (NOX4) [104]. Furthermore, Pelicano and colleagues have suggested that mitochondrial dysfunction may result in increased ROS production. This, in turn, could enhance the expression of the chemokine C-X-C motif 14 (CXCL14) via the AP-1 signaling pathway, thereby increasing cytosolic calcium levels and improving cell motility [105]. Finally, the stimulation of Kruppel-like factor 9 (Klf9) by ROS on nuclear factor erythroid 2-related factor 2 (Nrf2) leads to the activation of ERK1/2 and an increase in ROS generation in cancer cells [106,107,108]. Collectively, these studies highlight the pivotal role of ROS in various aspects of tumorigenesis and metastasis, including cell proliferation, survival, and motility.

## 4. Edaravone

### 4.1. Mechanisms of Action

Edaravone is chemically known as 3-methyl-1-phenyl-2-pyrazoline-5-one and is assigned the code number MCI-186. It is a colorless transparent liquid with a modest acidity, indicated by its acid dissociation constant (pKa) of 7.0. Due to its lipophilic formulation, when administered intravenously, edaravone can easily penetrate the blood–brain barrier (BBB) and is minimally affected by P-gp efflux [109]. Additionally, edaravone exhibits keto–enol tautomerism, which significantly influences its action as a scavenger of radicals, thus endowing it with antioxidant properties [110,111]. More specifically, edaravone acts as a scavenger for peroxyl radicals and peroxynitrite, providing cytoprotection and neuroprotection against oxidative stress. By neutralizing ROS, such as the hydroxyl radical, peroxyl radical, hydrogen peroxide, peroxynitrite, and other compounds that contribute to neuronal degeneration, edaravone safeguards neurons in the brain and spinal cord, thereby protecting against neurological damage and motor neuron death [110,111]. Interestingly, there is evidence that edaravone protects neurons from oxidative stress by altering mitochondrial activity, but the exact mechanism of action is still unknown [112]. It helps restore mitochondrial function both in laboratory settings (in vitro) and animal models (in vivo), preventing cellular toxicity. Notably, edaravone treatment has been shown to inhibit the loss of mitochondrial membrane potential, downregulate the mitochondrial apoptosis pathway [113], prevent swollen mitochondrial morphology [114], restore mitochondrial complex I activity [115], and decrease ROS production in paraquat-treated A549 cells [116]. Edaravone is also able to reduce lipid peroxidation just as effectively as ascorbic acid and vitamin E, two well-known antioxidants [117]. Regarding the antioxidant activity of edaravone as a scavenger of peroxyl radicals, more research is being carried out to investigate the role of pH. It has been observed that the consumption rate of edaravone increases with rising pH levels. Moreover, Ohara and collaborators showed that the scavenging activity of edaravone becomes more pronounced at higher pH levels [118].

Edaravone also modulates the activities of Nrf2 [119]. Nrf2 is known to play a role in protecting against oxidative stress through its activation of anti-inflammatory and antioxidative effects, regulating the expression of antioxidant and detoxification enzymes [120,121]. Regarding edaravone, some studies demonstrated its protective effects results due to the activation of the Nrf2/HO-1 pathway, which reduces oxidative damage through Nrf2 pathway activation and acts as a defense mechanism against cell apoptosis [119,122]. Moreover, it has been reported that activating the Nrf2/HO-1 signaling pathway with edaravone enhances the integrity and stability of the BBB and can serve as a key target for therapeutic treatment in cases of cerebral infarction [119]. Another study investigated the neuroprotective effects of edaravone on the hippocampus of rats with kainate-induced epilepsy via the Nrf2/HO-1 signaling pathway. According to Liu et al., edaravone mitigated the downregulation of mRNA and protein expression levels of Nrf2 and HO-1 induced by kainite [123].

In addition to neutralizing free radicals and modulating Nrf2 activities, edaravone also possesses anti-inflammatory properties by regulating NFκB and suppressing the release of pro-inflammatory cytokines, which are often dysregulated in cancer. Specifically, edaravone inhibits the translocation of the NFκB transcription factor from the cytoplasm to the nucleus while simultaneously activating the expression of Nrf2 to regulate downstream antioxidant genes, as mentioned earlier [124,125]. Additionally, edaravone reduces the levels of several pro-inflammatory cytokines/chemokines, including IL-1β, IL-6, IL-10, and TNF-α, as demonstrated in different experimental models and humans [123,126,127].

### 4.2. Anticancer Effects

**Figure 3:**
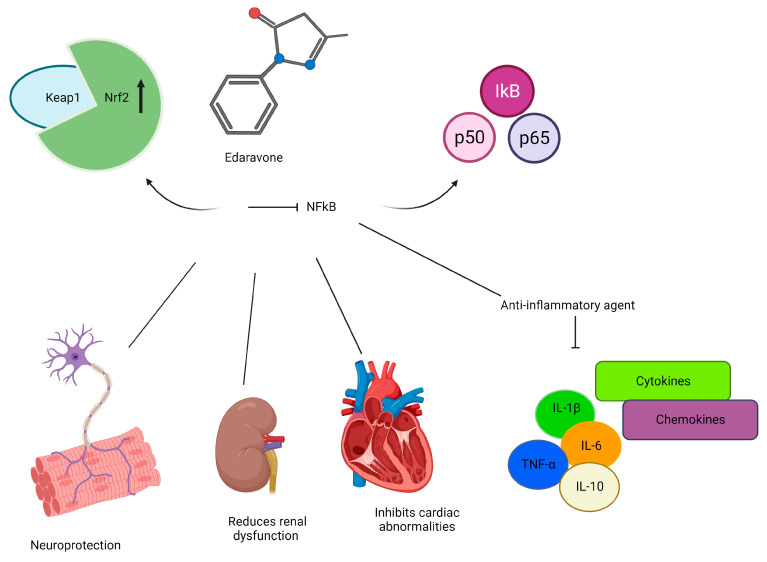
Schematic summary of edaravone proprieties and its potential effects in cancer pathology. Created with BioRender.com (accessed on 17 January 2024).

The potential anticancer effects of edaravone, whether used alone or in combination with other treatments, have been explored in both laboratory (in vitro) and animal (in vivo) models of tumors. Several studies have reported a modest level of anticancer activity for edaravone [128,129,130,131] (Figure 3). Edaravone was shown to dose-dependently inhibit tumor growth in various human cancer cell lines, including hepatocarcinoma HepG2, mesothelioma MSTO-211H, gastric carcinoma TMK-1, and breast carcinoma MCF-7 [131]. This effect was achieved through its influence on the signaling of EGFR and by arresting the cell cycle. However, this effect was limited and primarily observed at high concentrations, with minimal impact on cell viability [131]. As edaravone itself is a weak cytotoxic drug, it was also evaluated in combination with the pterin derivative DFP [129,130]. It has been demonstrated that combining DFP with edaravone makes the cell more permeable and causes significantly greater intracellular ROS generation and cell death than DFP alone [130]. In murine colon cancer models, one study investigated whether edaravone could enhance the anticancer effects of irinotecan CPT-11, a potent inhibitor of the enzyme topoisomerase I used in the treatment of solid tumors. The research aimed to assess whether edaravone could improve the effectiveness of CPT-11 by investigating its ability to inhibit tumor growth. However, this drug was also able to activate the transcription factor NFκB, which represents one mechanism of tumor resistance to apoptosis, induced by chemotherapy and radiotherapy [132]. As a result, the authors suggested that blocking NFκB activation with edaravone can shift the balance of cell survival/death toward apoptosis. In vitro results demonstrated that edaravone inhibits the NFκB activation induced by SN38, the active metabolite of CTP-11, further increasing apoptosis. Moreover, combining edaravone and CTP-11 treatment reduced tumor growth and the number of pulmonary metastases more than CTP-11 alone [128]. These findings suggested that the combination of these two drugs may represent a novel strategy for the treatment of primary and metastatic colon cancer by enhancing apoptosis via inhibiting NFκB activation.

Recently, Duarte and collaborators evaluated the anticancer profile of edaravone both alone and in combination with chemotherapeutic drugs in MCF-7 breast and HT-29 colon cancer cell models. However, data showed that edaravone has no cytotoxic effects in both cell lines when used alone or in combination with paclitaxel and doxorubicin [133].

### 4.3. Cytoprotective Effects against Conventional Cancer Therapies

Recent studies also revealed that edaravone displays a significant cytoprotective role against side effects induced by conventional cancer therapies, which affect patients’ quality of life. One of the notable effects is its ability to reduce cardiotoxicity, renal failure, and neurotoxicity induced by chemotherapeutic agents, which can limit the effectiveness of cancer treatment. For instance, studies have shown that edaravone can prevent doxorubicin-induced cardiac deterioration without affecting the anticancer effect of daunorubicin [134]. In animal studies, pre-treatment with edaravone alleviated doxorubicin-associated cardiac abnormalities [135]. Additionally, several studies investigated a possible cytoprotective effect of edaravone against cisplatin-induced renal damage, neurotoxicity, and ototoxicity. The combination of cisplatin and edaravone reduced renal dysfunction and renal tubular injury in both in vitro and in vivo models [136,137,138,139,140]. In animal models, edaravone treatment improved interstitial fibrosis, tubule injury, and cell infiltration. It has also been explored as an antioxidant agent to mitigate cisplatin-induced neurobehavioral impairments [138]. It has been shown to reduce renal dysfunction, renal tubular injury, cyst development, and mitochondrial damage caused by cisplatin. In animal models, edaravone treatment improved interstitial fibrosis, tubule injury, and cell infiltration. It has also been explored as an antioxidant agent to mitigate cisplatin-induced neurobehavioral impairments [140]. The authors demonstrated that treatment with edaravone increases Nrf2/HO-1 expression and inhibits the cisplatin-induced NFκB activation, resulting in decreased neurobehavioral and cognitive deficits [141]. Other two studies reported a protective role of edaravone against cisplatin-mediated ototoxicity both in an auditory cell line [142] and a zebrafish model [143] by preventing apoptosis and limiting ROS production [144]. In addition to anthracyclines and cisplatin, edaravone was also evaluated in combination with the anticancer drug cyclophosphamide, a chemotherapeutic agent with severe neurotoxic side effects. Research has shown that administering edaravone and cyclophosphamide together can ameliorate the behavioral and histopathological changes induced by the chemotherapeutic agent [145]. Furthermore, the combined treatment can prevent cyclophosphamide chemotherapy-induced alopecia, improving cancer patients’ quality of life [146].

In addition to reducing toxicity from chemotherapeutic agents, edaravone has been investigated for its ability to alleviate side effects induced by radiotherapy. At high doses, edaravone has demonstrated radioprotective properties by suppressing X-ray-induced apoptosis [147] and preventing phosphorylated histone H2AX (γH2AX) foci formation [148]. However, at low concentrations, it can enhance X-ray-induced apoptosis in certain cell lines with wild-type p53 status [149]. Edaravone has also shown protective effects on human neural stem cells, promoting their survival and differentiation after irradiation [150]. In vivo studies have demonstrated dose-dependent and injection time-dependent radioprotection by edaravone [151]. Additionally, in a clinical trial involving patients with nasopharyngeal carcinoma, the combined administration of edaravone and corticosteroids improved neurologic symptoms and reduced edema area, suggesting potential benefits in reducing radiation-induced brain necrosis [152]. Moreover, edaravone has been found to protect against radiation-induced oral mucositis, commonly observed in patients undergoing radiotherapy for head and neck cancer [153].

Furthermore, there is evidence to suggest that edaravone may have potential use in decreasing the incidence of thyroid dysfunctions, such as thyroiditis [154], which are common among cancer patients receiving immunotherapy treatment with immune checkpoint inhibitors, particularly anti-PD-1 antibodies [155,156].

The main effects of edaravone identified by in vitro and in vivo studies are summarized in Table 2.

## 5. Conclusions and Future Perspectives

Despite significant advancements in oncology research, cancer remains a leading cause of mortality worldwide and imposes a substantial financial burden on patients and healthcare systems. To address this challenge, drug repurposing has emerged as a promising strategy in the quest for effective and durable cancer treatments. This approach holds the potential for overcoming drug resistance and enabling precise cancer therapies.

Edaravone, a free scavenger currently used for the treatment of acute cerebral infarction and ALS, has also been investigated in the context of cancer due to the association between cancer and oxidative stress. However, when it comes to its anticancer effects, it exhibits only modest or limited anti-proliferative properties, whether used alone or in combination with other drugs. Nevertheless, intriguingly, co-treatment of this drug with CTP-11 has shown potential in reducing tumor growth and the occurrence of pulmonary metastases in a colon cancer model.

On the other hand, edaravone demonstrates significant cytoprotective effects against conventional cancer therapies, including chemotherapy, radiotherapy, and immunotherapy. It can serve as a valuable tool in reducing cardiotoxicity, renal failure, ototoxicity, and neurotoxicity induced by various chemotherapeutic agents, as well as mitigating side effects caused by radiotherapy. Furthermore, the use of edaravone shows promise in reducing autoimmune thyroiditis induced by immunotherapy treatment.

However, the applicability of edaravone in cancer treatment shows some limitations. Major concerns are its extremely low aqueous solubility and limited bioavailability, which hamper its intestinal absorption and necessitate the use of high-dose parenteral administration [157]. This route of drug delivery can be challenging for cancer patients, whereas oral administration would be preferable and may help to improve patients’ quality of life by avoiding infusion-related stress, the risk of infusion-related infections or extravasations, infusion-related discomfort, and the need for further administration visits. To address this challenge, novel oral and sublingual tablet formulations of edaravone are currently under investigation to improve their oral bioavailability, yielding encouraging results [158,159]. Another concern in utilizing this drug for cancer treatment may be the risk of drug resistance by several mechanisms, including increased DNA repair ability, altered drug metabolism and targets, inhibited apoptosis, and enhanced efflux of drugs. The development of the nanoparticle-based delivery of edaravone may provide an approach to overcome drug resistance by increasing drug permeability and circulation time, retention, and intracellular accumulation.

In conclusion, the data discussed in this review suggest that edaravone may have potential applications in supporting cancer treatment. Nevertheless, further clinical studies are required to validate the observed effects of edaravone in cellular and animal models and to overcome the above-mentioned limitations of its use in clinical settings.

## Figures and Tables

**Table 1:** A summarizing table of conventional cancer therapies and their effects.

Kind of Therapy	Beneficial Effects	Side Effects
Surgery	Tumor removal, symptom relief, local control	Increased pain, recurrence, inappropriateness for some cancer types, limitations in case of metastases
Chemotherapy	Broad coverage, apoptosis of tumor cells, utilization in various cancer stages	Damage to healthy cells, possible ineffectiveness, impact on quality of life
Radiotherapy	Localized treatment, preserving the functionality of nearby sites, treatment flexibility	Potential impairment of target organ functionality, limitations in case of metastases
Hematopoietic stem cell transplantation	Cell replacement, curative for some blood cancers, potential long-term cure	Complications and risks, limited number of compatible donors, extended recovery period, risk of rejection
Hormone therapy	Targeted tumor cells, various administration modes, maintenance treatment	Variable response, hormonal side effects, potential long-term issues, limited efficacy in advanced stages
Immunotherapy	Target specificity, long-lasting response, application to various cancers, minimal side effects	Risk of autoimmune reactions, possible insufficiency in response, variable response among patients
Targeted therapy	Precision in targeting, efficacy, fewer side effects, quick response, specific cancer types	Limitations in non-targeted tumors, genetic complexity, specific side effects
Precision therapy	Treatment personalization, good efficacy, minimal side effects, potential to improve quality of life	Development of resistance, genetic complexity, limitations in non-mutated tumors, variable response

**Table 2:** In vitro and in vivo summary of the main effects of edaravone.

The Actions of Edaravone In Vitro	References	In Vivo	References
Inhibition of HepG2, MSTO-211H, TMK-1, and MCF-7 tumor growth in a dose-dependent manner	[105,106,107,108]	Cytoprotective: reduces cardiotoxicity, renal failure, and neurotoxicity	[111,112,113,114,115,116,117]
Weak cytotoxic drug	[106,107]	Antioxidant	[115]
Blocking NFκB activation	[109]	Limiting ROS production	[121]
Has an anticancer effect in MCF-7 and HT-29 in combination with paclitaxel or doxorubicin	[110]	Radioprotective	[124,125,127,128]
		Reduces the occurrence of thyroid dysfunctions	[131]

## Data Availability

Not applicable.
